# Assessment of seasonality and normalization techniques for wastewater-based surveillance in Ontario, Canada

**DOI:** 10.3389/fpubh.2023.1186525

**Published:** 2023-08-30

**Authors:** Hadi A. Dhiyebi, Joud Abu Farah, Heather Ikert, Nivetha Srikanthan, Samina Hayat, Leslie M. Bragg, Asim Qasim, Mark Payne, Linda Kaleis, Caitlyn Paget, Dominika Celmer-Repin, Arianne Folkema, Stephen Drew, Robert Delatolla, John P. Giesy, Mark R. Servos

**Affiliations:** ^1^Department of Biology, University of Waterloo, Waterloo, ON, Canada; ^2^Regional Municipality of York, Newmarket, ON, Canada; ^3^Regional Municipality of Waterloo, Waterloo, ON, Canada; ^4^Department of Civil Engineering, University of Ottawa, Ottawa, ON, Canada; ^5^Department of Biomedical Sciences and Toxicology Centre, University of Saskatchewan, Saskatoon, SK, Canada; ^6^Department of Environmental Science, Baylor University, Waco, TX, United States

**Keywords:** fecal indicators, normalization, seasonality, SARS-CoV-2, PMMoV, crAssphage

## Abstract

**Introduction:**

Wastewater-based surveillance is at the forefront of monitoring for community prevalence of COVID-19, however, continued uncertainty exists regarding the use of fecal indicators for normalization of the SARS-CoV-2 virus in wastewater. Using three communities in Ontario, sampled from 2021–2023, the seasonality of a viral fecal indicator (pepper mild mottle virus, PMMoV) and the utility of normalization of data to improve correlations with clinical cases was examined.

**Methods:**

Wastewater samples from Warden, the Humber Air Management Facility (AMF), and Kitchener were analyzed for SARS-CoV-2, PMMoV, and crAssphage. The seasonality of PMMoV and flow rates were examined and compared by Season-Trend-Loess decomposition analysis. The effects of normalization using PMMoV, crAssphage, and flow rates were analyzed by comparing the correlations to clinical cases by episode date (CBED) during 2021.

**Results:**

Seasonal analysis demonstrated that PMMoV had similar trends at Humber AMF and Kitchener with peaks in January and April 2022 and low concentrations (troughs) in the summer months. Warden had similar trends but was more sporadic between the peaks and troughs for PMMoV concentrations. Flow demonstrated similar trends but was not correlated to PMMoV concentrations at Humber AMF and was very weak at Kitchener (*r* = 0.12). Despite the differences among the sewersheds, unnormalized SARS-CoV-2 (raw N1–N2) concentration in wastewater (*n* = 99–191) was strongly correlated to the CBED in the communities (*r* = 0.620–0.854) during 2021. Additionally, normalization with PMMoV did not improve the correlations at Warden and significantly reduced the correlations at Humber AMF and Kitchener. Flow normalization (*n* = 99–191) at Humber AMF and Kitchener and crAssphage normalization (*n* = 29–57) correlations at all three sites were not significantly different from raw N1–N2 correlations with CBED.

**Discussion:**

Differences in seasonal trends in viral biomarkers caused by differences in sewershed characteristics (flow, input, etc.) may play a role in determining how effective normalization may be for improving correlations (or not). This study highlights the importance of assessing the influence of viral fecal indicators on normalized SARS-CoV-2 or other viruses of concern. Fecal indicators used to normalize the target of interest may help or hinder establishing trends with clinical outcomes of interest in wastewater-based surveillance and needs to be considered carefully across seasons and sites.

## Introduction

Due to the COVID-19 pandemic, there has been increased interest in wastewater-based surveillance (WBS) to monitor community prevalence of SARS-CoV-2, with the majority of studies taking place in high-income countries (1). A number of these studies have compared the raw SARS-CoV-2 concentration to clinical metrics (e.g., active cases, new cases, or hospitalizations) [see Li et al. (2) for a review]. However, wastewater systems are very diverse with contrasting infrastructure (3) even within regional settings. The characteristics of the sewer (e.g., sanitary or combined with stormwater, network dynamics, residence time) can influence the fate of fecal matter and viral pathogens, such as SARS-CoV-2, and pose challenges for the interpretation of WBS data. Industrial or commercial inputs and inflow and infiltration (I/I) events can also cause challenges to WBS due to inhibition, dilution, or scouring of settled material (4). Normalization of the viral signal to fecal indicators or flow is frequently done to partially address these concerns. However, normalization of the viral signal is often done without consideration of the complexity of the wastewater or the sewershed and may lead to additional variability. A better understanding of the variability of parameters used to normalize the viral signal and the relationship to key clinical indicators is needed to ensure WBS is optimized for each community.

Several wastewater parameters have been used as fecal indicators for normalization of SARS-CoV-2 in many studies including the pepper mild mottle virus (PMMoV) and cross-assemblage phage (crAssphage). PMMoV is a highly abundant RNA virus found on plants that are commonly found in human diets. It is consistently found in human feces and therefore has been recommended and applied widely as a fecal contamination indicator (5–7). crAssphage is a DNA-based bacteriophage that has been proposed as another human fecal contamination indicator as it is highly associated with human feces, is abundant and ubiquitous in wastewater (8, 9), and has been used in previous studies to normalize viral signals (10). Commonly measured wastewater parameters such as flow, NH_3_, total Kjeldahl nitrogen (TKN), total suspended solids (TSS), carbonaceous biological oxygen demand (CBOD), pH, and biological oxygen demand (BOD) have also been proposed and used for normalization of viral signals (11, 12). Other normalization techniques using chemical tracers such as artificial sweetener (acesulfame), caffeine, and its metabolite paraxanthine as well as human metabolites (creatine, 5-hydroxyindoleacetic acid) have also been used with various success (13, 14).

In some sewersheds, normalization of SARS-CoV-2 using fecal indicators has been shown to improve correlations with clinical metrics (15, 16). However, others have shown normalization by fecal indicators has minimal improvement or negatively impacts correlations between the SARS-CoV-2 wastewater measurements and clinical metrics (13, 17, 18). Many factors may influence the patterns of each fecal indicator, including seasonal patterns in sewer flow (e.g., I/I), and sources. For example, PMMoV may be influenced by the seasonal availability of produce or consumption patterns in diets (6). There is therefore a need to investigate why normalizations with fecal indicators seem to be useful in some sewersheds and not others, including the influence of seasonal differences. As WBS will undoubtedly continue to be a widely applied tool, understanding and reducing the uncertainty regarding the value of normalizing will be important for future surveillance programs. This study examines the value of viral signal normalization by assessing wastewater measurements from three communities in Ontario over an extended period during the COVID-19 pandemic (January 2021–February 2023). The seasonal variability in the fecal biomarker PMMoV is examined and the utility of using biomarkers (i.e., PMMoV, crAssphage) and flow to improve the correlations between SARS-CoV-2 wastewater estimates and clinical cases by episode date (CBED) is assessed across seasons and sites.

## Methods

### Wastewater sampling and locations

Twenty-four-hour time-weighted composite influent wastewater samples were collected at the Kitchener municipal wastewater treatment plant (Kitchener, Ontario, Canada) and at a well at the Humber Air Management Facility (AMF) pumping station that collects wastewater from the west side of Vaughan, Regional Municipality of York (York Region). A third site (Warden main sewer line in York Region) was grab sampled due to the depth of the sewer. Three grab samples were collected at the same time each sampling day and were combined, mixed, and then sub-sampled. The 2021 populations served in the three wastewater sampling sites were approximately 256,000, 105,000, and 659,000 at the Kitchener, Humber AMF, and Warden sewersheds, respectively. Samples were stored in pre-cleaned HDPE containers, kept at 4°C, and transported to the University of Waterloo (Waterloo, Ontario, Canada) for nucleic acid concentration, extraction, and qPCR analysis. As part of a surveillance program, the data were analyzed and normally reported within 3 days of receiving the samples. Wastewater parameters (i.e., TSS, pH, TKN, CBOD, BOD, and NH_3_) and flow rates were provided by the public works department of the respective regions and are summarized in Appendix Table S1.

### Nucleic acid concentration, extraction, and quantification via RT-qPCR

A modified PEG-precipitation/centrifugation method was used for each wastewater sample as described in Dhiyebi et al. (19). Briefly, a 40 mL wastewater sample was added to a 50 mL Falcon centrifuge tube with PEG 8000 (4 g) and NaCl (0.9 g). The sample was shaken on ice for 2 h and left to settle at 4°C overnight. The sample was then centrifuged at 12,000 *g* for 1.5 h at 4°C with no brake to concentrate the virus into the solids with the supernatant discarded. A second centrifugation step at 12,000 *g* for 15 min at 4°C with no brake was used to solidify the pellet and discard any remaining supernatant. Nucleic acids were extracted and purified from the solids using Power Microbiome Kit (QIAGEN, United States) following the manufacturer’s protocol with up to 250 mg (wet weight) of the pellet resuspended in a TRIzol/PM1 solution, respectively, using an automated QIAcube (QIAGEN, United States). The DNase step was excluded from extraction to allow for the measurement of crAssphage, a DNA virus. The nucleic acids were eluted in 100 μL nuclease-free water. Extracted nucleic acids then underwent one-step RT-qPCR for SARS-CoV-2 N1, N2 gene targets (20) and PMMoV (21). A subset of samples was later analyzed by qPCR to measure crAssphage [CPQ056; (9)]. The PCR assays, cycling conditions, and performances are described in Appendix Tables S2–S4, respectively. Each sample was also assessed for inhibition (reverse transcription and PCR) and each plate had standard curves, positive control, and non-template controls (NTCs) as recommended by the MIQE guidelines (22). Samples that were inhibited were removed from the dataset prior to analysis and accounted for less than 10% (46/477) of the total samples analyzed. As there was a strong correlation between N1 and N2 concentrations (Pearson’s *r* = 0.858) and it is essentially measuring the same virus, the SARS-CoV-2 concentrations were presented as the mean of N1 and N2 (N1–N2) to reduce variability and improve the estimate. PCR data is presented as log2 concentration (gene copies/mL).

### Assessing seasonal trends

Seasonality was assessed with a Seasonal-Trend-Loess (STL) decomposition (23) with the “timetk” R package (version 2.8.2). STL decomposition is a robust method to filter a time series into 3 components: Seasonal, Trend, and Remainder using LOESS (23). This method allows for determining any temporal patterns (seasonal or trend) within a timeseries dataset and minimizes the effects of outliers. The frequency was defined as 1 week intervals and the trend was defined as 3 months intervals. PMMoV seasonality was analyzed from all sites for the entire study period for each site. For Humber AMF and Kitchener, the sample dates ranged from January 2021 to February 2023, while for Warden the sampling began in April 2021. Flow rates from Humber AMF and Kitchener (minimum of three measurements a week) were available, but Warden flow rates were not available from the main sewer line (a modelled estimate of 150 ML/d was provided by York Region). Flow rates were used to determine the possible impact of rain events and snowmelt (e.g., storm water/infiltration) on wastewater endpoints. Data are presented as monthly boxplots and STL decomposition plots (i.e., observed, trend).

### Clinical cases correlation comparison

SARS-CoV-2 copies/mL was determined as the mean of N1 and N2 (N1–N2) in each sample. All viral concentrations were log2(*x*) transformed and CBED was transformed as log(*x* + 1) prior to analysis for normality. The Pearson’s correlation (pairwise) was performed the transformed SARS-CoV-2 concentrations (raw or PMMoV normalized) and CBED between January and 1 December 2021. During this period, the number of daily clinical tests (mean ± standard deviation) conducted in the province was 34,585 ± 15,040 (24). This timeframe was chosen as clinical testing was conducted at a high level in Ontario until the emergence of the Omicron variant overwhelmed the testing capacity and testing eligibility was changed, resulting in a bias that underestimated clinical cases after late December 2021 (19). The relationships between clinical metrics (i.e., clinical cases or hospitalizations) and wastewater may also be confounded by the emergence of variants (e.g., Delta) and changes in the vaccination status of the population and test-seeking behaviors (25–27). The emergence of the Delta variant in the mid-summer of 2021 may have partially changed the wastewater ratio in some Ontario communities but not others (25). In the US, the appearance of Delta may have only weakly altered the relationship to COVID-19 incidence rates at other sites (28). The entire period prior to the appearance of Omicron was therefore used for the comparison between the raw and normalized correlations to CBED. A time-step comparison of the correlations between CBED and wastewater for up to 10 days lag was also conducted. A subset of samples was later analyzed for crAssphage to compare the crAssphage normalization technique directly to the PMMoV normalized or raw signal (*n* = 32, 31, and 57 for Warden, Humber AMF and Kitchener respectively).

The “cocor” R package (version 1.1.4) was then used to compare whether these correlations between clinical cases by episode date (CBED) and the raw or normalized (PMMoV or crAssphage) SARS-CoV-2 concentrations were significantly different from one another (29). This package offers a wide range of statistical tests to compare correlations (29). The comparisons were between two overlapping correlations based on dependent groups. The correlations were overlapping since CBED was used in all correlation comparisons and dependent as the same N1–N2 concentration was used for both the raw and normalized values (i.e., the same wastewater sample). The correlations were deemed significantly different (*α* = 0.05) if the confidence interval did not include zero (30).

## Results

### Trend/seasonality analysis

**Figure 1:**
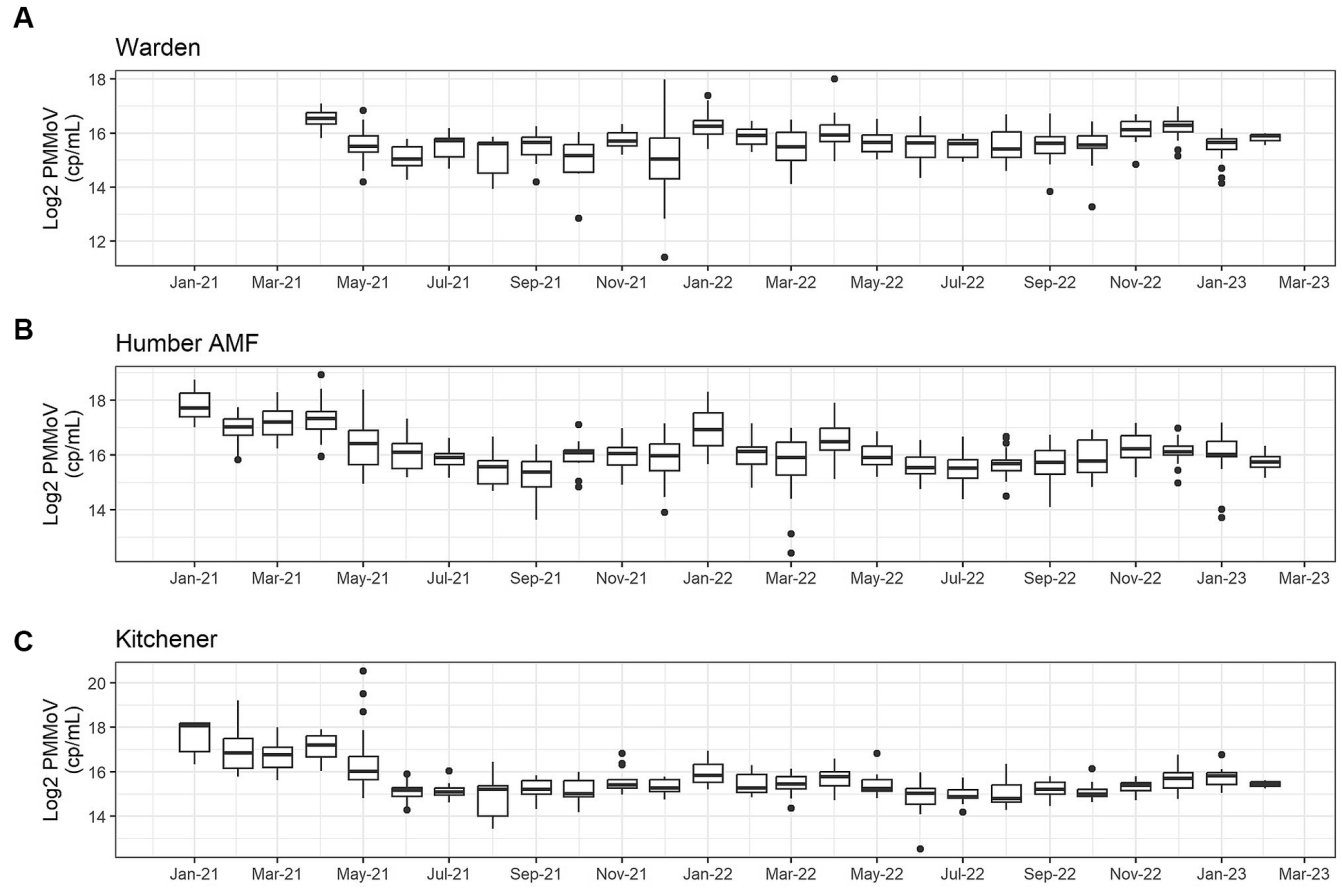
Monthly log2 PMMoV concentrations (copies/mL) from January 2021-Februrary 2023 at the Warden **(A)**, Humber AMF **(B)**, and Kitchener **(C)** wastewater sampling sites.

**Figure 2:**
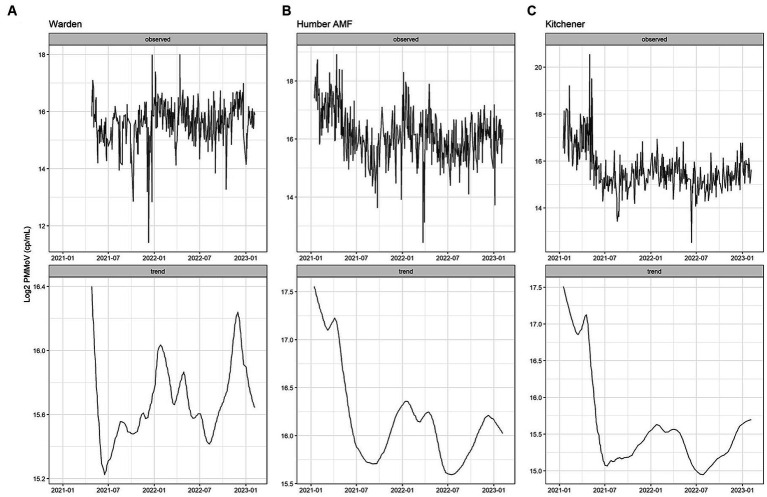
PMMoV Seasonal Trend Loess (STL) decomposition plots for the Warden **(A)**, Humber AMF **(B)**, and Kitchener **(C)** wastewater sampling sites. The frequency was set as 1 week and the trend length was 3 months.

**Figure 3:**
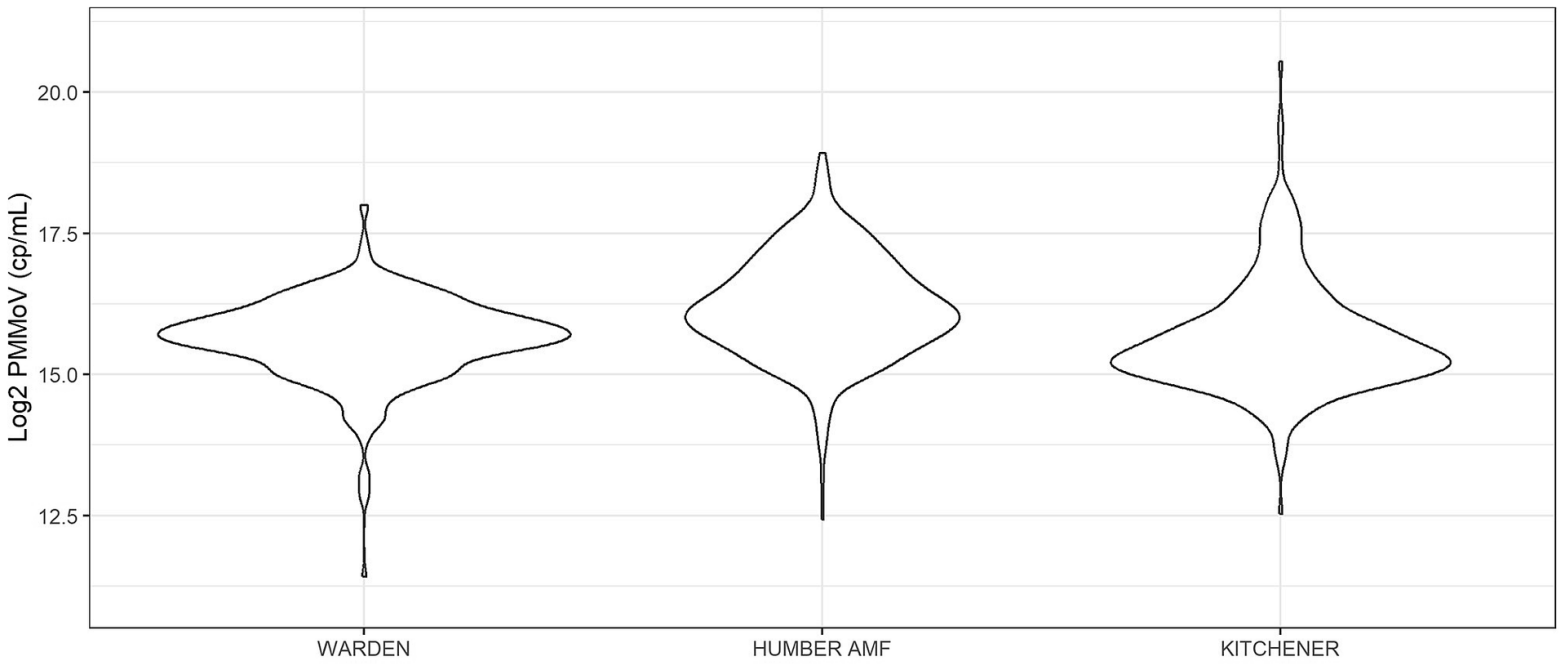
Violin distribution plots for the Warden, Humber AMF, and Kitchener wastewater sampling sites. Warden collections were from April 2021 to February 2023, and Humber AMF and Kitchener collections were from January 2021 to February 2023.

At all sites, PMMoV concentrations were consistently high between January and May 2021 (Figures 1, 2). PMMoV concentrations were the lowest between the summer months and early fall (June–October). There were two main peaks at Humber AMF and Kitchener for PMMoV concentrations in January and April 2022 with monthly median values of 15.8 log2 copies/mL for both months in Kitchener and 16.9 and 16.5 log2 copies/mL, respectively, for Humber AMF. At Warden, PMMoV concentrations appeared to follow the general trend of the other two sites with peaks in the late fall and early winter months (November to February) and lower concentrations in the summer months. However, PMMoV concentrations were more sporadic between the peaks and troughs at Warden due to the lower interquartile range (IQR; 0.75 log2 cp/mL) compared to the other two sites (IQR = 0.98–1.11 log2 cp/mL; Appendix Table S5). This variability in PMMoV concentrations over the entire period in Warden compared to the other two sites is further demonstrated in the violin distribution plots (Figure 3), where there are more concentrations that have higher probability densities compared to Humber AMF and Kitchener.

**Figure 4:**
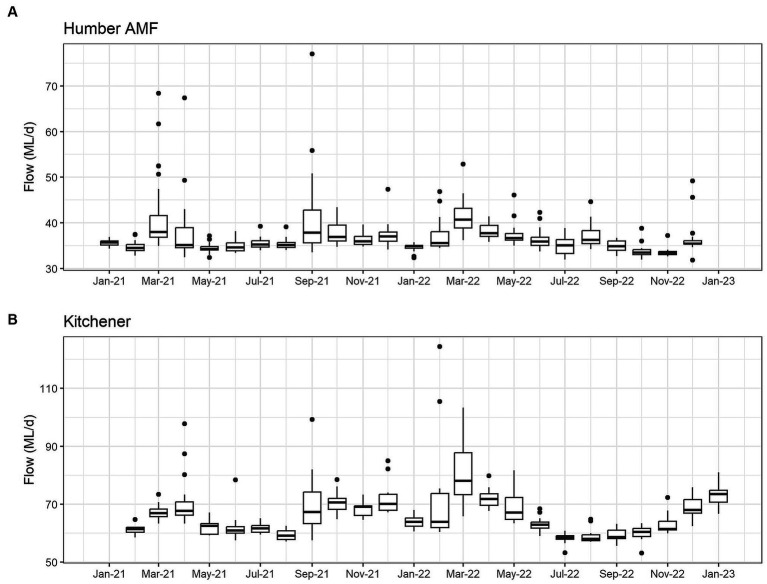
Monthly wastewater flow (ML/d) from January 2021 to February 2023 at the Humber AMF **(A)** and Kitchener **(B)** wastewater sampling sites.

**Figure 5:**
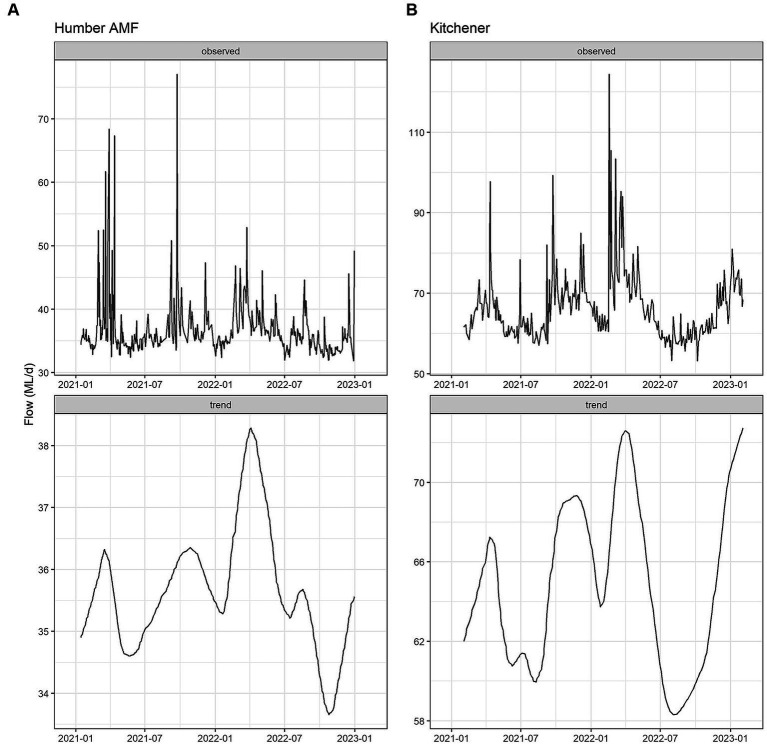
Flow Seasonal Trend Loess (STL) decomposition plots for the Humber AMF **(A)** and Kitchener **(B)** wastewater sampling sites. The frequency was set as 1 week and the trend length was 3 months.

The seasonal flow trends in Humber AMF and Kitchener were higher in the spring and fall seasons with the late summer months having the lowest flow. Specifically, in March 2022, both sites had the highest median flow at the sampling location with Humber AMF having a median of 40.7 ML/d and Kitchener having a median of 78.1 ML/d. In general, the peak flow seasons in Humber AMF were March–April and August–September, whereas in Kitchener the changes in flow rates seemed to be more gradual with a few exceptions (Figures 4, 5). There was no correlation and a weak correlation (*r* = 0.12) between flow rates and PMMoV concentrations at Humber AMF and Kitchener, respectively. conveys the intended meaning.

### Clinical cases correlation comparisons

#### Raw N1–N2 and PMMoV normalization compared to clinical cases in 2021

**Figure 6:**
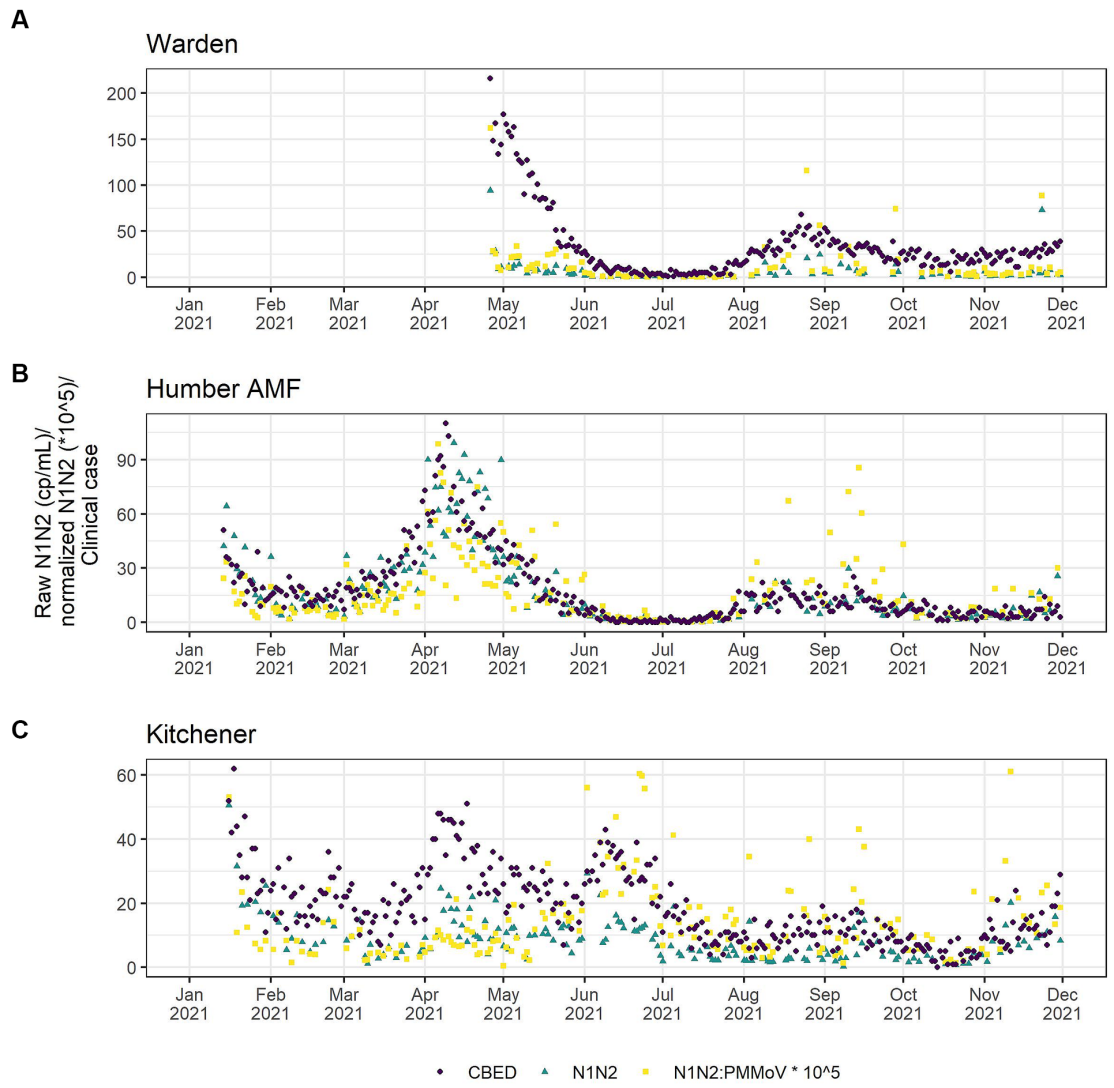
Clinical cases by episode date (purple circle) and wastewater SARS-CoV-2 in raw (green triangle) and PMMoV normalized (yellow square) N1–N2 concentrations from the Warden **(A)**, Humber AMF **(B)** and Kitchener **(C)** Wastewater sampling sites. Warden sample dates were from April to December 2021. Humber AMF and Kitchener sample dates were from January to December 2021.

A comparison of correlations using a time lag between the CBED and wastewater signals determined that it did not impact the relative differences between the raw and normalized relationships (Appendix Table S6). Additionally, there were no significant differences (*α* = 0.05) in the correlation estimates between the highest correlation with lag and correlations with no lag (Appendix Table S7), so only the data without consideration of a lag is presented further for clarity. At Humber AMF and Kitchener, the raw mean N1 and N2 copies/mL (N1–N2) correlations with CBED were significantly better than the normalized (N1–N2/PMMoV) correlations with CBED (Figure 6; Table 1). At Kitchener, the normalized correlation (Pearson’s *r*) was substantially lower than the raw N1–N2 (non-normalized) correlation (0.167 compared to 0.620, Table 1). However, at Warden, there were no significant differences between the PMMoV normalized correlation and the raw N1–N2 correlation for the entire study period. The flow normalization correlations were not significantly different from the raw correlations with *r* = 0.856 for Humber AMF and *r* = 0.613 for Kitchener.

**Table 1:** Pearson correlation coefficients (*r*) between cases by episode date and wastewater measure between January 15th and December 1st, 2021 at the Warden, Humber AMF, and Kitchener wastewater sampling sites.

Site (*n*)	Raw N1–N2	PMMoV normalized N1–N2
Warden (99, 98)	0.781	0.696
Humber AMF (191)	0.854^*^	0.702
Kitchener (175)	0.620^*^	0.167

Comparisons were done on two overlapping correlations based on dependent groups with the cocor package using 95% confidence intervals (30). An ^*^ indicates significantly different correlations (*p* = 0.05).

#### Subset of data with crAssphage normalization

**Figure 7:**
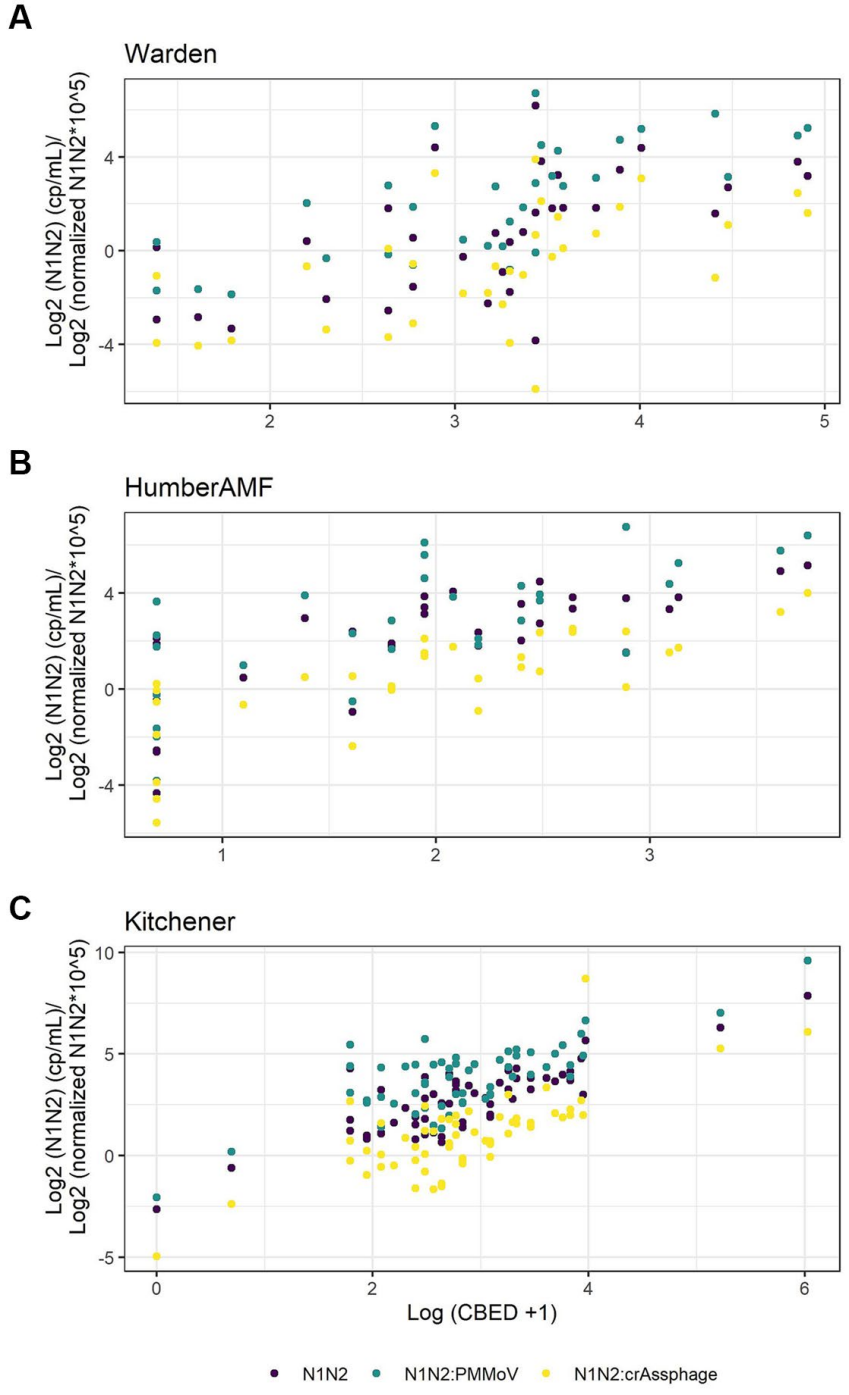
Scatterplots of new cases by episode date (CBED) and the raw (N1–N2), crAssphage normalized (N1–N2/crAssphage), and pepper mild mottle virus normalized (N1–N2/PMMoV) wastewater concentrations at Warden (*n* = 32, **A**), Humber AMF (*n* = 29–31, **B**) and Kitchener (*n* = 57, **C**).

For Kitchener, the correlation between the raw concentration of SARS-CoV-2 and cases by episode date was significantly better than the PMMoV normalization and the crAssphage normalization (Figure 7; Table 2). However, in the subset data, the correlation of clinical cases with the PMMoV normalized concentration was significantly higher than using the raw concentration or the crAssphage normalized concentrations at Warden. Interestingly, at Humber AMF, there were no significant differences between the raw concentration correlation and both the PMMoV and the crAssphage normalization correlations (Table 2).

**Table 2:** Pearson correlation coefficients (*r*) between cases by episode date and wastewater measure on the subset of data from the Warden, Humber AMF, and Kitchener wastewater sampling sites.

Site (*n*)	Raw N1–N2	PMMoV normalized N1–N2	CrAssphage normalized N1–N2
Warden (32)	0.608^a^	0.712^b^	0.553^a^
Humber AMF (29–31)	0.726	0.696	0.767
Kitchener (57)	0.833^a^	0.766^b^	0.751^b^

Comparisons were done on two overlapping correlations based on dependent groups with the cocor package using 95% confidence intervals (30). Letters indicate significant differences between correlations (*p* = 0.05).

## Discussion

PMMoV normalization did not improve the correlations between wastewater SARS-CoV-2 concentrations and clinical cases at three Ontario sites sampled in 2021. The raw N1–N2 concentrations had significantly better correlations with clinical cases at both Kitchener and Humber AMF compared to the PMMoV-normalized correlations with clinical cases. At Warden, the raw and PMMoV-normalized correlations were similar. This may be due to the different characteristics of this sewershed, such as higher flow (nearly double the other two sites), potentially longer travel time (up to 24 h), and a larger population covered. The lack of improved correlations with clinical cases using PMMoV normalization is in agreement with other studies that also did not report a consistent advantage in normalizing the SARS-CoV-2 concentration (16–18, 31). Maal-Bared et al. (17) found that PMMoV normalization only improved correlation to clinical cases in 2 of 12 sites in Alberta, Canada, and Duvallet et al. (32) found that normalization of 55 sites in the United States was inconsistent in improving correlations. At all sites, crAssphage normalization did not improve correlations to clinical cases compared to the raw N1–N2 correlations using a subset of the data (i.e., Figure 7). However, using this subset of data from Warden, the PMMoV normalization correlation to clinical cases was slightly better than the raw N1–N2 correlation (95% confidence interval of the difference, 0.268 to 0.006), which is in contrast to the larger dataset where there was no difference. This demonstrates the complexity of wastewater-based surveillance and how difficult it is to establish these relationships, especially with small datasets. Normalization of the SARS-CoV-2 signal with PMMoV may be an advantage in some sites or times possibly by accounting for variation in flow, fecal content, or sampling technique, but great care needs to be taken with the interpretation of normalized data. It may be dependent on the source of the sample (pipe, influent, sludge), and characterizing over multiple seasons would greatly improve our insights into how these biomarkers might be used effectively.

Numerous studies have shown that fecal biomarker normalization (PMMoV or crAssphage) is very site-specific in terms of improving correlations with clinical cases (16, 33, 34). Normalization might help in comparisons among sites, however, within sewersheds normalization might not assist in enhancing the relationships with clinical cases (35). The differences observed across sites indicate that in some cases normalization with fecal indicators could play an important role in improving WBS trends, but this needs to be assessed on a site-specific basis using statistical approaches such as STL analysis and the correlation comparisons described in this study.

The usefulness and appropriateness of normalization remains a topic of considerable debate. The limitations of using fecal viral biomarkers, such as PMMoV and crAssphage, include the uncertainty associated with relative fecal shedding rates of viruses and their fate in the sewershed. However, recent studies on individual shedding rates of SARS-CoV-2 (36) and fecal biomarkers (37) are addressing this knowledge gap although there remains considerable uncertainty. Additional studies on the fate of viruses once in the sewershed are needed as this is important for the interpretation of the surveillance results. In addition, it has been shown that viruses partition differently under various conditions (38, 39). Flow has been used to normalize SARS-CoV-2 as well (10, 19), however, in this study flow normalization correlations were not significantly different from unnormalized correlations. A limitation of flow normalization is that flow measurements may be unobtainable at some locations due to site characteristics (such as the Warden site in this study). In addition, rapid access to flow data, if available, may also be a limitation. Despite all these limitations, normalization and specifically measuring fecal biomarkers can be effective as a quality check of wastewater samples and lab processes and therefore has additional value (40).

Despite the weak or lack of correlation between PMMoV and flow rates at Humber AMF and Kitchener, the PMMoV concentrations were generally higher during the higher flow seasons. This suggests that environmental factors, such as rain events, were not diluting fecal material but in fact, the high flow events were scouring settled materials in the sewer and increasing the concentration of PMMoV at the collection site. PMMoV tends to partition primarily to the supernatant fraction, likely associated with very fine particles or colloids of the wastewater samples even after centrifugation at 12,000 *g,* however, a substantial proportion (~15%) of PMMoV is still associated with solids (39). The fate of PMMoV in the sewer may therefore differ from SARS-CoV-2 which is more evenly partitioned between the solids and supernatant (39). If wastewater volume increases because of environmental effects (i.e., rain events, snow melt) it would be anticipated that the viral concentration of the biomarker would decrease as wastewater gets diluted. However, this pattern was not observed in the three Ontario sites in the current study. Additionally, even though the temperature in the sewershed does not fluctuate as much as air temperature (e.g., Warden wastewater ranged from 10.4°C to 20.2°C), it may still play a significant role in the prevalence of PMMoV. This adds additional uncertainty when assessing the suitability of PMMoV as a fecal indicator and might be one of the contributions to the variability in the normalized SARS-CoV-2 signal at some sites.

The higher variability of PMMoV in Warden might be due in part to the collection approach applied at this site. Grab samples, even when well mixed from large flows, may not be as representative as 24 h composite sampling. In the case of SAR-CoV-2 wastewater sampling, Bivins et al. (41) demonstrated temporal variability in concentration during the day. This variability may lead to lower detection rates of grab samples, especially in small sewersheds (42, 43). However, others have seen good concurrence for the detection of SARS-CoV-2 in wastewater when directly comparing grabs and composite samples (44–46). Grab samples from the Warden site represent a major wastewater flow and large population which results in batch samples having some variability, but still relatively consistent PMMoV over time. Additional studies comparing the two sampling approaches from a single site over an extended period of time would be helpful to understand the impact of the sampling approach. In situations where there is considerable sample or temporal variability, normalization may still provide an advantage.

The goal of early studies using fecal indicators, such as PMMoV and crAssphage, was for the detection of fecal contamination of surface/source water, therefore a good indicator would be highly abundant in wastewater to increase the sensitivity of detection (6). However, for the normalization of respiratory or enteric viruses, the goal is to have an indicator that reflects the inputs and fate of fecal material in the sewershed so that variations in the sewershed, environmental conditions, and flow can be accounted for. This poses a challenge for the selection of a robust endpoint that can be used for normalization to improve the correlation of the viral signal to clinical endpoints of interest. As PMMoV and crAssphage are present at much higher levels (10^5^ to 10^9^ copies/mL) in wastewater compared to SARS-CoV-2 concentrations (usually less than 10^3^ copies/mL) considerable variation can be added when normalizing. Ideally, the viral signal would be normalized with a marker that has similar properties and fate in the sewershed, can be reliably detected, and is strongly correlated with the source of the viral signal of interest. Currently, there are no ideal indicators available to universally normalize viral signals, such as SARS-CoV-2, in wastewater. Identification of reliable and validated indicators (e.g., viruses, bacteria, human genes, or chemicals) or groupings of indicators, that can be used to normalize viral signals in wastewater will greatly enhance the application of WBS and our ability to correlate wastewater signals to clinical endpoints of concern.

## Data availability statement

The data has been deposited in the Federated Research Data repository (FRDR) with DOI: 10.20383/102.0702.

## Author contributions

HD and MS contributed to the conception and original writing of the manuscript. HD, MS, NS, SH, and HI contributed to the methodology, investigation, data curation, and project administration. LK and JA contributed to the methodology and investigation of the manuscript. LB contributed to the project administration. JG and MS provided the funding acquisitions for this project. AQ, MP, CP, DC-R, AF, SD, and RD contributed to the conception of this manuscript. All authors contributed to the article and approved the submitted version.

## Funding

This research was funded from the Ontario Ministry of the Environment, Conservation and Parks: Wastewater Surveillance Initiative (Grant No. TPA 2021-02-1-1564736554). In addition, this research was in part funded by the Canada First Research Excellence Fund under the Global Water Futures Program (Grant # 410295/419205). This research was also supported by an NSERC Discovery Grant (MS RP-2017-03816); the Canada Research Chairs program (MS and JG); and Distinguished Visiting Professorship in the Department of Environmental Sciences, Baylor University in Waco, TX (JG).

## Conflict of interest

The authors declare that the research was conducted in the absence of any commercial or financial relationships that could be construed as a potential conflict of interest.

## Publisher’s note

All claims expressed in this article are solely those of the authors and do not necessarily represent those of their affiliated organizations, or those of the publisher, the editors and the reviewers. Any product that may be evaluated in this article, or claim that may be made by its manufacturer, is not guaranteed or endorsed by the publisher.

## References

[1] Naughton CC Roman FA Jr Alvarado AGF Tariqi AQ Deeming MA Kadonsky KF. Show us the data: global COVID-19 wastewater monitoring efforts, equity, and gaps. FEMS Microbes. (2023) 4:xtad003. doi: 10.1093/femsmc/xtad003, PMID: 37333436 PMC10117741

[2] Li X Zhang S Sherchan S Orive G Lertxundi U Haramoto E. Correlation between SARS-CoV-2 RNA concentration in wastewater and COVID-19 cases in community: a systematic review and meta-analysis. J Hazard Mater. (2023) 441:129848. doi: 10.1016/j.jhazmat.2022.129848, PMID: 36067562 PMC9420035

[3] von Sperling M. Wastewater characteristics, treatment and disposal (2015). 9781780402086 p Available at: http://iwaponline.com/ebooks/book-pdf/1075/wio9781780402086.pdf.

[4] Bertels X Demeyer P van den Bogaert S Boogaerts T van Nuijs ALN Delputte P. Factors influencing SARS-CoV-2 RNA concentrations in wastewater up to the sampling stage: a systematic review. Sci Total Environ. (2022) 820:153290. doi: 10.1016/j.scitotenv.2022.153290, PMID: 35066048 PMC8772136

[5] Rosario K Symonds EM Sinigalliano C Stewart J Breitbart M. Pepper mild mottle virus as an indicator of fecal pollution. Appl Environ Microbiol. (2009) 75:7261–7. doi: 10.1128/AEM.00410-09, PMID: 19767474 PMC2786529

[6] Symonds EM Rosario K Breitbart M. Pepper mild mottle virus: agricultural menace turned effective tool for microbial water quality monitoring and assessing (waste) water treatment technologies. PLoS Pathog. (2019) 15:1–7. doi: 10.1371/journal.ppat.1007639 PMC6472819 30998781

[7] Kitajima M Sassi HP Torrey JR. Pepper mild mottle virus as a water quality indicator. npj Clean Water. (2018) 1:19. doi: 10.1038/s41545-018-0019-5

[8] Stachler E Bibby K. Metagenomic evaluation of the highly abundant human gut bacteriophage CrAssphage for source tracking of human fecal pollution. Environ Sci Technol Lett. (2014) 1:405–9. doi: 10.1021/ez500266s

[9] Stachler E Kelty C Sivaganesan M Li X Bibby K Shanks OC. Quantitative CrAssphage PCR assays for human fecal pollution measurement. Environ Sci Technol. (2017) 51:9146–54. doi: 10.1021/acs.est.7b02703, PMID: 28700235 PMC7350147

[10] Langeveld J Schilperoort R Heijnen L Elsinga G Schapendonk CEM Fanoy E. Normalisation of SARS-CoV-2 concentrations in wastewater: the use of flow, electrical conductivity and crAssphage. Sci Total Environ. (2023) 865:161196. doi: 10.1016/j.scitotenv.2022.161196 36581271 PMC9791714

[11] Hoar C Li Y Silverman AI. Assessment of commonly measured wastewater parameters to estimate sewershed populations for use in wastewater-based epidemiology: insights into population dynamics in New York city during the COVID-19 pandemic. ACS ES T Water. (2022) 20:2014–24. doi: 10.1021/acsestwater.2c00052 37552716

[12] Hutchison JM Li Z Chang C-N Hiripitiyage Y Wittman M Sturm BSM. Improving correlation of wastewater SARS-CoV-2 gene copy numbers with COVID-19 public health cases using readily available biomarkers. FEMS Microbes. (2022) 3:xtac010. doi: 10.1093/femsmc/xtac010, PMID: 36118159 PMC9480869

[13] Xie Y Challis JK Oloye FF Asadi M Cantin J Brinkmann M. RNA in municipal wastewater reveals magnitudes of COVID-19 outbreaks across four waves driven by SARS-CoV-2 variants of concern. ACS ES T Water. (2022) 2:1852–62. doi: 10.1021/acsestwater.1c00349, PMID: 37552734

[14] Hsu S-Y Bayati M Li C Hsieh H-Y Belenchia A Klutts J. Biomarkers selection for population normalization in SARS-CoV-2 wastewater-based epidemiology. Water Res. (2022) 223:118985. doi: 10.1101/2022.03.14.22272359, PMID: 36030667 PMC9376872

[15] D’Aoust PM Mercier E Montpetit D Jia JJ Alexandrov I Neault N. Quantitative analysis of SARS-CoV-2 RNA from wastewater solids in communities with low COVID-19 incidence and prevalence. Water Res. (2021) 188:116560. doi: 10.1016/j.watres.2020.116560, PMID: 33137526 PMC7583624

[16] Nagarkar M Keely SP Jahne M Wheaton E Hart C Smith B. SARS-CoV-2 monitoring at three sewersheds of different scales and complexity demonstrates distinctive relationships between wastewater measurements and COVID-19 case data. Sci Total Environ. (2022) 816:151534. doi: 10.1016/j.scitotenv.2021.151534, PMID: 34780821 PMC8590472

[17] Maal-Bared R Qiu Y Li Q Gao T Hrudey SE Bhavanam S. Does normalization of SARS-CoV-2 concentrations by pepper mild mottle virus improve correlations and lead time between wastewater surveillance and clinical data in Alberta (Canada): comparing twelve SARS-CoV-2 normalization approaches. Sci Total Environ. (2023) 856:158964. doi: 10.1016/j.scitotenv.2022.158964, PMID: 36167131 PMC9508694

[18] Feng S Roguet A McClary-Gutierrez JS Newton RJ Kloczko N Meiman JG. Evaluation of sampling, analysis, and normalization methods for SARS-CoV-2 concentrations in wastewater to assess COVID-19 burdens in Wisconsin communities. ACS ES T Water. (2021) 1:1955–65. doi: 10.1021/acsestwater.1c00160

[19] Dhiyebi HA Cheng L Varia M Atanas K Srikanthan N Hayat S. Estimation of COVID-19 case incidence during the omicron outbreaks based on SARS-CoV-2 wastewater load in previous waves, Peel region, Canada. Emerg Infect Dis. (2023) 29:1580–8. doi: 10.3201/eid2908.221580 37379513 PMC10370834

[20] Center for Disease Control. Research use only 2019-novel coronavirus (2019-nCoV) real-time RT-PCR primers and probes. CDC’s diagnostic test for COVID-19 only and supplies (2020) 2019–2020. Available at: https://www.cdc.gov/coronavirus/2019-ncov/lab/virus-requests.html

[21] Zhang T Breitbart M Lee WH Run J-Q Wei CL Soh SWL. RNA viral community in human feces: prevalence of plant pathogenic viruses. PLoS Biol. (2006) 4:e3. doi: 10.1371/journal.pbio.0040003 16336043 PMC1310650

[22] Bustin SA Benes V Garson JA Hellemans J Huggett J Kubista M. The MIQE guidelines: minimum information for publication of quantitative real-time PCR experiments. Clin Chem. (2009) 55:611–22. doi: 10.1373/clinchem.2008.112797 19246619

[23] Cleveland RB Cleveland WS McRae JE Terpenning I. STL: a seasonal-trend decomposition procedure based on loess (with discussion). J Off Stat. (1990) 6:3–73. Available at: http://cs.wellesley.edu/~cs315/Papers/stl statistical model.pdf

[24] Government of Ontario. Status of COVID-19 cases in Ontario. (2020). Available at: https://data.ontario.ca/en/dataset/status-of-covid-19-cases-in-ontario/resource/ed270bb8-340b-41f9-a7c6-e8ef587e6d11 (Accessed June 1, 2023).

[25] D’Aoust PM Tian X Towhid ST Xiao A Mercier E Hegazy N. Wastewater to clinical case (WC) ratio of COVID-19 identifies insufficient clinical testing, onset of new variants of concern and population immunity in urban communities. Sci Total Environ. (2022) 853:158547. doi: 10.1016/j.scitotenv.2022.158547, PMID: 36067855 PMC9444156

[26] Hegazy N Cowan A Aoust PMD Mercier É Towhid ST Jia J. Understanding the dynamic relation between wastewater SARS-CoV-2 signal and clinical metrics throughout the pandemic. Sci Total Environ. (2022) 853:158458. doi: 10.1016/j.scitotenv.2022.158458 36075428 PMC9444583

[27] Boehm AB Wolfe MK White BJ Hughes B Duong D. Divergence of wastewater SARS-CoV-2 and reported laboratory-confirmed COVID-19 incident case data coincident with wide-spread availability of at-home COVID-19 antigen tests. *medRxiv* (2023). Available at: https://www.medrxiv.org/content/10.1101/2023.02.09.23285716v1. [Epub ahead of preprint] 10.7717/peerj.15631 PMC10312197 37397016

[28] Schoen ME Wolfe MK Li L Duong D White BJ Hughes B. SARS-CoV-2 RNA wastewater settled solids surveillance frequency and impact on predicted COVID-19 incidence using a distributed lag model. ACS ES T Water. (2022) 2:2167–74. doi: 10.1021/acsestwater.2c00074, PMID: 36380770 PMC9092194

[29] Diedenhofen B Musch J. Cocor: a comprehensive solution for the statistical comparison of correlations. PLoS One. (2015) 10:1–12. doi: 10.1371/journal.pone.0121945 PMC4383486 25835001

[30] Zou GY. Toward using confidence intervals to compare correlations. Psychol Methods. (2008) 12:399–413. doi: 10.1037/1082-989X.12.4.399 18179351

[31] Greenwald HD Kennedy LC Hinkle A Whitney ON Fan VB Crits-Christoph A. Tools for interpretation of wastewater SARS-CoV-2 temporal and spatial trends demonstrated with data collected in the San Francisco Bay Area. Water Res X. (2021) 12:100111. doi: 10.1016/j.wroa.2021.100111, PMID: 34373850 PMC8325558

[32] Duvallet C Wu F Mcelroy KA Imakaev M Endo N Xiao A. Nationwide trends in COVID-19 cases and SARS-CoV-2 RNA wastewater concentrations in the United States. ACS ES T Water. (2022) 2:1899–909. doi: 10.1021/acsestwater.1c00434, PMID: 36380771 PMC9092192

[33] Mitranescu A Uchaikina A Kau AS Stange C Ho J Tiehm A. Wastewater-based epidemiology for SARS-CoV-2 biomarkers: evaluation of normalization methods in small and large communities in southern Germany. ACS ES T Water. (2022) 2:2460–70. doi: 10.1021/acsestwater.2c00306, PMID: 37552738

[34] Kim S Kennedy LC Wolfe MK Criddle CS Duong DH Topol A. SARS-CoV-2 RNA is enriched by orders of magnitude in primary settled solids relative to liquid wastewater at publicly owned treatment works. Environ Sci Water Res Technol. (2022) 8:757–70. doi: 10.1039/D1EW00826A, PMID: 35433013 PMC8969789

[35] Wolfe MK Archana A Catoe D Coffman MM Dorevich S Graham KE. Scaling of SARS-CoV-2 RNA in settled solids from multiple wastewater treatment plants to compare incidence rates of laboratory-confirmed COVID-19 in their sewersheds. Environ Sci Technol Lett. (2021) 8:398–404. doi: 10.1039/D1EW00826A, PMID: 37566351

[36] Natarajan A Zlitni S Brooks EF Vance SE Dahlen A Hedlin H. Gastrointestinal symptoms and fecal shedding of SARS-CoV-2 RNA suggest prolonged gastrointestinal infection. Med. (2022) 3:371–387.e9. doi: 10.1016/j.medj.2022.04.001, PMID: 35434682 PMC9005383

[37] Arts PJ Daniel Kelly J Midgley CM Anglin K Lu S Andino R. Longitudinal and quantitative fecal shedding dynamics of SARS-CoV-2, 1 pepper mild mottle virus and CrAssphage. mSphere. (2023) e00132–23. doi: 10.1128/msphere.00132-23 PMC10506459 37338211

[38] Roldan-Hernandez L Boehm AB. Adsorption of respiratory syncytial virus (RSV), rhinovirus, SARS-CoV-2, and F^+^ bacteriophage MS2 RNA onto wastewater solids from raw wastewater. *bioRxiv* (2023) Available at: https://www.biorxiv.org/content/10.1101/2023.05.04.539429v1. [Epub ahead of preprint] 10.1021/acs.est.3c03376 PMC10501194 37647137

[39] Breadner PR Dhiyebi HA Fattahi A Srikanthan N Hayat S Aucoin MG. A comparative analysis of the partitioning behaviour of SARS-CoV-2 RNA in liquid and solid fractions of wastewater. Sci Total Environ. (2023) 895:165095. doi: 10.1016/j.scitotenv.2023.165095, PMID: 37355124 PMC10287177

[40] Torii S Oishi W Zhu Y Thakali O Malla B Yu Z. Comparison of five polyethylene glycol precipitation procedures for the RT-qPCR based recovery of murine hepatitis virus, bacteriophage phi6, and pepper mild mottle virus as a surrogate for SARS-CoV-2 from wastewater. Sci Total Environ. (2022) 807:150722. doi: 10.1016/j.scitotenv.2021.150722, PMID: 34610400 PMC8487407

[41] Bivins A North D Wu Z Shaffer M Ahmed W Bibby K. Within- and between-day variability of SARS-CoV-2 RNA in municipal wastewater during periods of varying COVID-19 prevalence and positivity. ACS ES T Water. (2021) 1:2097–108. doi: 10.1021/acsestwater.1c00178

[42] George AD Kaya D Layton BA Bailey K Mansell S Kelly C. Impact of sampling type, frequency, and scale of the collection system on SARS-CoV-2 quantification fidelity. Environ Sci Technol Lett. (2022) 9:160–5. doi: 10.1021/acs.estlett.1c00882, PMID: 37566370

[43] Nguyen Quoc B Saingam P RedCorn R Carter JA Jain T Candry P. Case study: impact of diurnal variations and stormwater dilution on SARS-CoV-2 RNA signal intensity at neighborhood scale wastewater pumping stations. ACS ES T Water. (2022) 2:1964–75. doi: 10.1021/acsestwater.2c00016, PMID: 37552740

[44] Kmush BL Monk D Green H Sachs DA Zeng T Larsen DA. Comparability of 24-hour composite and grab samples for detection of SARS-2-CoV RNA in wastewater. FEMS Microbes. (2022) 3:xtac017. doi: 10.1093/femsmc/xtac017/6609772, PMID: 37332496 PMC10117866

[45] Augusto MR Claro ICM Siqueira AK Sousa GS Caldereiro CR Duran AFA. Sampling strategies for wastewater surveillance: evaluating the variability of SARS-COV-2 RNA concentration in composite and grab samples. J Environ Chem Eng. (2022) 10:107478. doi: 10.1016/j.jece.2022.107478, PMID: 35251931 PMC8882035

[46] Curtis K Keeling D Yetka K Larson A Gonzalez R. Wastewater SARS-CoV-2 concentration and loading variability from grab and 24-hour composite samples. *medRxiv* (2020). Available at: https://www.medrxiv.org/content/10.1101/2020.07.10.20150607v2. [Epub ahead of preprint]

