# Supplementary material for: Assessment of seasonality and normalization techniques for wastewater-based surveillance in Ontario, Canada
Source: Front Public Health. 2023 Aug 30;11:1186525. doi: 10.3389/fpubh.2023.1186525 (PMC10499178; doi:10.3389/fpubh.2023.1186525)
Supplement: Supplementary file 1 [file Data_Sheet_1.docx]

**Appendix**

**Methods**

PCR conditions and controls:

The standard curve consisted of a minimum of 5 concentrations. A positive control was added to each plate to assess inter-plate variability. The positive controls used were approximately 90 copies/reaction for SARS-CoV-2 targets (N1, N2) and 56,250 copies/reaction for Pepper mild mottle virus. Each plate also had no reverse transcriptase control (NRT) as well as no template controls (NTC). Standard curves efficiency were between 90-110% with r^2^>0.98 as per the MIQE guidelines (Bustin *et al.*, 2009) and is summarized in Appendix Table 4.

A spike-in DNA (Zebrafish –gBlock^TM^, Integrated DNA Technologies, USA) or RNA (RNA MS2 Phage, Cat# 2033-18, USBiological Life sciences, USA) was used to assess inhibition. These two targets were chosen as they were not expected to be found in the wastewater sample extracts. The spike-in was added directly to the assay mastermix, which consisted of Taqpath one-step GC (Thermofisher, ###), primers and probes (see Appendix Table 2), mixed briefly and plated. Nuclease free water (5 µL) was used as a positive control, i.e. the expected Cq value associated with the spike-in. 5 µL of wastewater extract was added to sample wells in the PCR reaction. The wastewater sample was determined to be inhibited if the Cq was delayed by 1 Cq from the positive controls on the plate.

***Appendix Table 1:*** *Monthly mean wastewater parameters measured at the Warden, Humber AMF and Kitchener wastewater sampling sites. Modelled flow rate indicated by *.*

|  | Warden | | | Humber AMF | | | | | Kitchener | | | | | | |
| --- | --- | --- | --- | --- | --- | --- | --- | --- | --- | --- | --- | --- | --- | --- | --- |
| Month-Year | Flow* (ML/d) | TSS (mg/L) | pH | Flow (ML/d) | TSS (mg/L) | TKN (mg/L) | CBOD (mg/L) | BOD mg/L | Flow (ML/d) | TSS (mg/L) | TKN (mg/L) | CBOD (mg/L) | BOD mg/L | pH |  |
| Jan-21 | NA | NA | NA | 35,638.20 | 345.71 | NA | 332.86 | NA | NA | 146.20 | 44.48 | 148.75 | 165.75 | 7.62 |  |
| Feb-21 | NA | NA | NA | 35,023.18 | 345.88 | NA | 309.12 | NA | 61,822.03 | 268.71 | 51.28 | 174.75 | 190.75 | 7.62 |  |
| Mar-21 | NA | NA | NA | 41,630.45 | 338.92 | NA | 338.46 | NA | 70,770.62 | 201.69 | 56.00 | 189.40 | 213.60 | 7.70 |  |
| Apr-21 | 150,000 | 158.60 | 7.42 | 37,774.07 | 355.00 | NA | 322.69 | NA | 69,686.09 | 332.56 | 48.23 | 275.00 | 285.00 | 7.46 |  |
| May-21 | 150,000 | 213.75 | 7.50 | 34,399.29 | 283.85 | NA | 250.37 | NA | 62,308.75 | 370.47 | 59.23 | 290.00 | 302.50 | 7.47 |  |
| Jun-21 | 150,000 | 196.00 | 7.39 | 35,098.00 | 276.17 | NA | 310.00 | NA | 61,878.84 | 299.96 | 53.20 | 222.80 | 257.80 | 7.64 |  |
| Jul-21 | 150,000 | 164.75 | 7.19 | 35,547.65 | 202.19 | NA | 269.26 | NA | 60,994.72 | 265.56 | 61.03 | 220.25 | 251.00 | 7.64 |  |
| Aug-21 | 150,000 | 188.05 | 7.33 | 35,022.35 | 407.15 | NA | 312.59 | NA | 59,771.75 | 333.05 | 53.60 | 211.80 | 236.60 | 7.57 |  |
| Sep-21 | 150,000 | 166.33 | 7.34 | 39,451.17 | 421.88 | NA | 306.40 | 350.00 | 67,604.97 | 231.60 | 63.05 | 220.50 | 254.50 | 7.46 |  |
| Oct-21 | 150,000 | 152.20 | 7.29 | 38,094.65 | 338.50 | NA | 331.67 | 608.46 | 70,391.78 | 256.11 | 62.70 | 189.25 | 218.50 | 7.51 |  |
| Nov-21 | 150,000 | 155.05 | 7.35 | 36,198.00 | 321.05 | NA | 345.79 | 618.42 | 68,008.59 | 252.15 | 60.12 | 229.40 | 262.20 | 7.48 |  |
| Dec-21 | 150,000 | 150.14 | NA | 37,788.32 | 219.65 | NA | 254.71 | 485.88 | 72,769.27 | 242.62 | 51.30 | 229.25 | 257.25 | 7.57 |  |
| Jan-22 | 150,000 | 154.60 | NA | 34,723.00 | 176.81 | NA | 275.38 | 477.69 | 63,608.51 | 294.81 | 57.58 | 206.25 | 207.25 | 7.55 |  |
| Feb-22 | 150,000 | 157.36 | 7.49 | 38,619.89 | 354.44 | NA | 335.83 | 700.00 | 70,656.17 | 238.81 | 60.30 | 207.00 | 213.50 | 7.56 |  |
| Mar-22 | 150,000 | 167.00 | 7.54 | 41,391.94 | 216.57 | NA | 254.78 | NA | 81,513.30 | 257.68 | 45.76 | 178.40 | 191.00 | 7.52 |  |
| Apr-22 | 150,000 | 142.10 | 7.50 | 37,841.17 | 268.88 | 508.10 | 268.19 | NA | 71,977.25 | 274.63 | 54.25 | 227.75 | 230.75 | 7.54 |  |
| May-22 | 150,000 | 161.39 | 7.48 | 37,013.87 | 295.24 | 452.00 | 399.81 | NA | 68,755.42 | 240.00 | 63.95 | 214.50 | 232.50 | 7.40 |  |
| Jun-22 | 150,000 | 156.82 | 7.42 | 36,052.20 | NA | NA | NA | NA | 63,494.62 | 333.39 | 55.10 | 271.80 | 289.00 | 7.36 |  |
| Jul-22 | 150,000 | 208.00 | 7.35 | 78,079.00 | 405.71 | 611.76 | 535.88 | NA | 57,950.16 | 331.88 | 57.55 | 288.75 | 301.75 | 7.53 |  |
| Aug-22 | 150,000 | 201.91 | 7.37 | 37,038.90 | 383.20 | 550.00 | 388.00 | NA | 58,758.73 | 391.78 | 65.90 | 289.00 | 316.80 | 7.44 |  |
| Sep-22 | 150,000 | 237.58 | 7.34 | 34,721.57 | 368.57 | 800.67 | 424.62 | NA | 59,062.48 | 430.94 | 61.30 | 315.00 | 342.25 | 7.56 |  |
| Oct-22 | 150,000 | 252.79 | 7.37 | 33,732.16 | 333.46 | 580.38 | 395.38 | NA | 60,077.35 | 403.41 | 61.68 | 316.25 | 337.00 | 7.52 |  |
| Nov-22 | 150,000 | 196.05 | 7.35 | 33,648.50 | 534.00 | 418.57 | 384.21 | 707.83 | 63,388.87 | 403.00 | 57.52 | 267.20 | 277.00 | 7.59 |  |
| Dec-22 | 150,000 | 200.88 | 7.24 | 36,363.58 | 319.21 | 381.67 | 329.17 | 729.17 | 70,279.45 | 366.53 | 55.83 | 292.75 | 299.75 | 7.56 |  |
| Jan-23 | NA | NA | NA | NA | 348.30 | NA | 322.17 | NA | 72,782.46 | 271.61 | 53.80 | 250.25 | 271.75 | 7.67 |  |
| Feb-23 | NA | NA | NA | NA | NA | NA | NA | NA | 79,729.15 | 354.67 | 48.90 | 275.00 | 290.00 | 7.56 |  |

**Appendix Table 2:** PCR primer, probes and gBlock^TM^ sequences used in assays. Final concentrations for RT qPCR/ qPCR reactions.

| Target | Component | Final Concentration | Sequence 5’−3’ | Standard (supplier) |
| --- | --- | --- | --- | --- |
| N1  (Center for Disease Control, 2020) | Forward primer | 500 nM | GAC CCC AAA ATC AGC GAA AT | RNA Standard (EDX CoV019, Biorad) |
|  | Reverse primer | 500 nM | TCT GGT TAC TGC CAG TTG AAT CTG |  |
|  | Probe | 125 nM | ACC CCG CAT TAC GTT TGG TGG ACC (6-FAM / BHQ-1) |  |
| N2  (Center for Disease Control, 2020) | Forward primer | 500 nM | TTA CAA ACA TTG GCC GCA AA | RNA Standard (EDX CoV019, Biorad) |
|  | Reverse primer | 500 nM | GCG CGA CAT TCC GAA GAA |  |
|  | Probe | 125 nM | ACA ATT TGC CCC CAG CGC TTC AG (6-FAM / BHQ-1) |  |
| PMMoV  (Zhang *et al.*, 2006) | Forward primer | 400 nM | GAG TGG TTT GAC CTT AAC GTT GA | DNA standard (gBlock^TM^, IDT) |
|  | Reverse primer | 400 nM | TTG TCG GTT GCA ATG CAA GT |  |
|  | Probe | 125 nM | CCT ACC GAA GCA AAT G (Cy5 / BHQ-3) |  |
|  | Standard |  | AGG TAA TGG TAG CTG TGG TTT CAA ATG AGA GTG GTT TGA CCT TAA CGT TTG AGA GGC CTA CCG AAG CAA ATG TCG CAC TTG CAT TGC AAC CGA CAA TTA CAT CAA AGG AGG AAG GTT CGT TGA AG |  |
| HCoV-229E  (Vijgen *et al.*, 2005) | Forward primer | 400 nM | TTC CGA CGT GCT CGA ACT TT | DNA standard (gBlock^TM^, IDT) |
|  | Reverse primer | 400 nM | CCA ACA CGG TTG TGA CAG TGA |  |
|  | Probe | 200 nM | TCC TGA GGT CAA TGC A (6-FAM / BHQ-1) |  |
|  | Standard |  | GAT GTA CTT CGC AAA CAG TTT CAG ACT TTT CCG ACG TGC TCG AAC TTT TTG GGC ATG GAA TCC TGA GGT CAA TGC AAT CAC TGT CAC AAC CGT GTT GGG ACA GAC ATA CTA TCA ACC CAT TCA AC |  |
| Zebrafish  (in-house assay) | Forward primer | 900 nM | TGC GAA AAA CAC ACC CAG | DNA standard (gBlock^TM^, IDT) |
|  | Reverse primer | 900 nM | GGC AGA TGA AGA AGA AGG AAG |  |
|  | Probe | 250 nM | CAA TAC ACT ACA CCT CAG ACA TCT CAA CAG CA (6-FAM / BHQ-1) |  |
|  | Standard |  | ATG ACA AGC CTG CGA AAA ACA CAC CCA GTT TTA AAA ATC GCT AAT GAC GCA TTA GTT GAT TTG CCA ACG CCA CTA AAT ATT TCA GCG TGA TGA AAT TTT GGA TCT CTC CTT GGA TTA TGT CTT ATT ACA CAA ATT TTA ACA GGA CTA TTT TTA GCA ATA CAC TAC ACC TCA GAC ATC TCA ACA GCA TTT TCA TCT GTT GTG CAT ATT TGC CGA GAT GTA AAT TTC GGC TGA CTT ATT CGG AGC ATC CAT GCC AAT GGG GCT TCC TTC TTC TTC ATC TGC CTG TAT ATT CAC ATC GCC C |  |
| MS2 (Dreier *et al.*, 2005) | Forward primer | 500 nM | TGC TCG CGG ATA CCC G | RNA Standard (USBiological) |
|  | Reverse primer | 500 nM | AAC TTG CGT TCT CGA GCG AT |  |
|  | Probe | 125 nM | ACC TCG GGT TTC CGT CTT GCT CGT (HEX / BHQ-1) |  |
| *CrAssphage (CPQ_056)*  *(Stachler et al., 2017)* | Forward primer | 500 nM | CAG AAG TAC AAA CTC CTA AAA AAC GTA GAG | DNA standard (gBlock^TM^, IDT) |
|  | Reverse primer | 500 nM | GAT GAC CAA TAA ACA AGC CAT TAG C |  |
|  | Probe | 250 nM | [FAM] AAT AAC GAT TTA CGT GAT GTA AC [MGB] |  |
|  | Standard |  | CAG AAG TAC AAA CTC CTA AAA AAC GTA GAG GTA GAG GTA TTA ATA ACG ATT TAC GTG ATG TAA CTC GTA AAA AGT TTG ATG AAC GTA CTG ATT GTA ATA AAG CTA ATG GCT TGT TTA TTG GTC ATC |  |

**Appendix Table 3:** PCR cycling conditions.

| Target |  | N1/PMMoV | | N2/229E/Zebrafish/MS2/CrAssphage | |
| --- | --- | --- | --- | --- | --- |
|  | # of steps | Time | Temp (°C) | Time | Temp (°C) |
| UNG Incubation | 1x | 2 min | 25 | 2 min | 25 |
| Reverse Transcription | 1x | 15 min | 50 | 15 min | 50 |
| Polymerase Activation | 1x | 2 min | 95 | 2 min | 95 |
| Denature | 45x | 3 sec | 95 | 3 sec | 95 |
| Amplification |  | 30 sec | 55 | 30 sec | 60 |

**Appendix Table 4:** PCR standard curve efficiencies, y-intercept, r^2^ values and 95% Limit of detection (LOD_95%_) of standards used for SARS CoV-2 (N1, N2) and Pepper Mild Mottle Virus (PMMoV). The PMMoV LOD_95%_ was not determined as wastewater samples did not approach this limit.

| Target | Number of plates run (n) | Efficiency (mean ± SD) | Y-intercept (mean ± SD) | r² (mean ± SD) | LOD_95%_  (gene copies/  reaction) |
| --- | --- | --- | --- | --- | --- |
| N1 | 145 | 98.54 ± 5.99 | 39.92 ± 1.41 | 0.98 ± 0.01 | 2.12 |
| N2 | 148 | 97.68 ± 8.07 | 40.00 ± 1.27 | 0.99 ± 0.01 | 1.58 |
| PMMoV | 156 | 94.16 ± 3.89 | 42.66 ± 1.48 | 1.00 ± 0.003 | N/A |

**Appendix Table 5:** Summary of PMMoV concentrations (copies/mL) from Warden, Humber AMF and Kitchener.

| Site | Min | 1st quartile | Median | Mean | 3rd quartile | Max |
| --- | --- | --- | --- | --- | --- | --- |
| Warden | 2,725 | 40,412 | 53,087 | 56,822 | 67,809 | 262,230 |
| Humber AMF | 5,518 | 50,885 | 72,033 | 91,009 | 109,871 | 494,916 |
| Kitchener WWTP | 5,892 | 33,735 | 44,181 | 70,575 | 66,475 | 1,529,129 |

**Appendix Table 6:** Pearson correlation coefficients (r) between time-stepped cases by episode date (CBED) and wastewater measures (raw N1-N2 and PMMoV normalized N1-N2) between 15^th^ January and 1^st^ December 2021 at the Warden, Humber AMF and Kitchener wastewater sampling sites. Lag implies wastewater is in advance of CBED. Color scaled with red being the highest correlation and green being the lowest correlation.

|  | **Warden** | | **Humber AMF** | | **Kitchener** | |
| --- | --- | --- | --- | --- | --- | --- |
|  | **raw N1-N2** | **PMMoV normalized N1-N2** | **raw N1-N2** | **PMMoV normalized N1-N2** | **raw N1-N2** | **PMMoV normalized N1-N2** |
| raw N1-N2 | 1.000 | 0.950 | 1.000 | 0.876 | 1.000 | 0.522 |
| PMMoV normalized N1N2 | 0.950 | 1.000 | 0.876 | 1.000 | 0.522 | 1.000 |
| log1p cbed | 0.781 | 0.696 | 0.854 | 0.703 | 0.621 | 0.167 |
| log1p cbed lag1 | 0.806 | 0.729 | 0.856 | 0.690 | 0.620 | 0.200 |
| log1p cbed lag2 | 0.795 | 0.716 | 0.863 | 0.690 | 0.650 | 0.192 |
| log1p cbed lag3 | 0.781 | 0.705 | 0.837 | 0.693 | 0.613 | 0.142 |
| log1p cbed lag4 | 0.732 | 0.642 | 0.815 | 0.674 | 0.602 | 0.155 |
| log1p cbed lag5 | 0.756 | 0.674 | 0.821 | 0.664 | 0.602 | 0.144 |
| log1p cbed lag6 | 0.794 | 0.712 | 0.823 | 0.665 | 0.563 | 0.104 |
| log1p cbed lag7 | 0.750 | 0.645 | 0.799 | 0.632 | 0.537 | 0.094 |
| log1p cbed lag8 | 0.741 | 0.646 | 0.804 | 0.622 | 0.524 | 0.067 |
| log1p cbed lag9 | 0.713 | 0.608 | 0.785 | 0.609 | 0.576 | 0.070 |
| log1p cbed lag10 | 0.712 | 0.614 | 0.761 | 0.585 | 0.540 | 0.062 |

**Appendix Table 7:** Pearson’s correlations estimates and 95% confidence interval limits between cases by episode date (CBED) and wastewater measures with and without lags. Lead refers to the wastewater measurement and is in advance of CBED.

| **Site (lead time)** | **Raw/Normalized** | **cor (r)** | **lower CI (95%)** | **upper CI (95%)** |
| --- | --- | --- | --- | --- |
| Warden (no lead) | Raw | 0.781 | 0.690 | 0.847 |
| Warden (highest cor) lead 1 | Raw | 0.806 | 0.724 | 0.866 |
| Warden (no lead) | PMMoV Normalized | 0.696 | 0.578 | 0.786 |
| Warden (highest cor) lead 1 | PMMoV Normalized | 0.728 | 0.619 | 0.810 |
| Humber (no lead) | Raw | 0.854 | 0.810 | 0.888 |
| Humber AMF (highest cor) lead 2 | Raw | 0.862 | 0.821 | 0.895 |
| Humber AMF (highest cor) no lead | PMMoV Normalized | 0.702 | 0.622 | 0.768 |
| Kitchener (no lead) | Raw | 0.620 | 0.520 | 0.704 |
| Kitchener (highest cor) lead 2 | Raw | 0.650 | 0.555 | 0.728 |
| Kitchener (no lead) | PMMoV Normalized | 0.167 | 0.019 | 0.307 |
| Kitchener (highest cor) lead 1 | PMMoV Normalized | 0.199 | 0.052 | 0.337 |

**Appendix references**

Bustin, S.A. *et al.* (2009) ‘The MIQE guidelines: Minimum information for publication of quantitative real-time PCR experiments’, *Clinical Chemistry*, 55(4), pp. 611–622. Available at: https://doi.org/10.1373/clinchem.2008.112797.

Center for Disease Control (2020) *Research Use Only 2019-Novel Coronavirus (2019-nCoV) Real-time RT-PCR Primers and Probes*, *CDC’s Diagnostic Test for COVID-19 Only and Supplies*. Available at: https://www.cdc.gov/coronavirus/2019-ncov/lab/virus-requests.html.

Dreier, J., Störmer, M. and Kleesiek, K. (2005) ‘Use of bacteriophage MS2 as an internal control in viral reverse transcription-PCR assays’, *Journal of Clinical Microbiology*, 43(9), pp. 4551–4557. Available at: https://doi.org/10.1128/JCM.43.9.4551-4557.2005.

Stachler, E., Kelty, C., Sivaganesan, M., Li, X., Bibby, K., & Shanks, O. C. (2017). Quantitative CrAssphage PCR Assays for Human Fecal Pollution Measurement. *Environmental science & technology*, *51*(16), 9146–9154. https://doi.org/10.1021/acs.est.7b02703

Vijgen, L. *et al.* (2005) ‘Development of One-Step, Real-Time, Quantitative Reverse Transcriptase PCR Assays for Absolute Quantitation of Human Coronaviruses OC43 and 229E’, *Journal of Clinical Microbiology*, 43(11), pp. 5452–5456. Available at: https://doi.org/10.1128/JCM.43.11.5452-5456.2005.

Zhang, T. *et al.* (2006) ‘RNA viral community in human feces: prevalence of plant pathogenic viruses.’, *PLoS biology*. Edited by J. Dangl, 4(1), p. e3. Available at: https://doi.org/10.1371/journal.pbio.0040003.
